# Relationship between treatment regimens for visceral leishmaniasis and development of post-kala-azar dermal leishmaniasis and visceral leishmaniasis relapse: A cohort study from Bangladesh

**DOI:** 10.1371/journal.pntd.0007653

**Published:** 2019-08-15

**Authors:** Dinesh Mondal, Amresh Kumar, Abhijit Sharma, Moshtaq Mural Ahmed, Md. Golam Hasnain, Abdul Alim, M. Mamun Huda, Ridwanur Rahman, Jorge Alvar, Be-Nazir Ahmed, Rashidul Haque

**Affiliations:** 1 Nutrition and Clinical Services Division, International Centre For Diarrhoeal Disease Research, Bangladesh (icddr,b), 63 Shaheed Taj Uddin Ahmed Sarani, Mohakhali, Dhaka, Bangladesh; 2 PATH, Dr. Gopal Das Bhawan, New Delhi, India; 3 Department of Medicine, Shaheed Suhrawardy Medical College, Sher-E-Bangla Nagar, Dhaka, Bangladesh & Universal Medical College Research Centre, Mohakhali, Dhaka, Bangldesh; 4 Drugs for Neglected Diseases *initiative*, Chemin Louis-Dunant, Geneva, Switzerland; 5 Disease Control Unit, Directorate General of Health Services, Ministry of Health and Family Welfare of Bangladesh, Mohakhali, Dhaka, Bangladesh; 6 Infectious Disease Division, International Centre For Diarrhoeal Disease Research, Bangladesh (icddr,b), Shaheed Taj Uddin Ahmed Sarani, Mohakhali, Dhaka, Bangladesh; National Institute of Allergy and Infectious Diseases, UNITED STATES

## Abstract

**Background:**

We investigated the relationship of treatment regimens for visceral leishmaniasis (VL) with post-kala-azar dermal leishmaniasis (PKDL) and visceral leishmaniasis relapse (VLR) development.

**Methods:**

Study subjects included cohorts of patients cured of VL since treatment with monotherapy by sodium stibogluconate (SSG), miltefosine (MF), paromomycin intramuscular injection (PMIM), liposomal amphotericin B [AmBisome (AMB)] in a single dose (SDAMB) and in multidose (MDAMB), and combination therapies by AMB+PMIM, AMB+MF, and PMIM+MF. Follow up period was 4 years after treatment. Cohorts were prospective except SSG (retrospective) and MF (partially retrospective). We compared incidence proportion and rate in 100-person-4year of PKDL and VLR by treatment regimens using univariate and multivariate analysis.

**Findings:**

974 of 984 enrolled participants completed the study. Overall incidence proportion for PKDL and VLR was 12.3% (95% CI, 10.4%–14.5%) and 7.0% (95% CI, 5.6%–8.8%) respectively. The incidence rate (95% CI) of PKDL and VLR was 14.0 (8.6–22.7) and 7.6 (4.1–14.7) accordingly. SSG cohort had the lowest incidence rate of PKDL at 3.0 (1.3–7.3) and VLR at 1.8 (0.6–5.6), followed by MDAMB at 8.2 (4.3–15.7) for PKDL and at 2.7 (0.9–8.4) for VLR.

**Interpretation:**

Development of PKDL and VLR is related with treatment regimens for VL. SSG and MDAMB resulted in less incidence of PKDL and VLR compared to other treatment regimens. MDAMB should be considered for VL as a first step for prevention of PKDL and VLR since SSG is highly toxic and not recommended for VL.

## Author summary

Post-kala-azar Dermal Leishmaniasis (PKDL), a sequale of visceral leishmaniasis (VL), and reappearance VL (visceral leishmaniasis relapse, VLR) are intra-epidemic reservoirs of VL and threats control of VL in long run. Currently there is no strategy for prevention of PKDL and VLR. If a relationship between treatment for VL and development of PKDL and VLR is there, and then selection of proper treatment regimen for VL should prevent PKDL and VLR. So far no study has been carried out to investigate the relationship between treatment regimens for VL and development of PKDL and VLR. The study demonstrated that multi-dose liposomal amphotericin B (AmBisome) defined as MDAMB herein for VL results in least PKDL and VLR among all existing and recommended by WHO treatment regimens for VL. We recommend adaptation of MDAMB in the national visceral leishmaniasis elimination program for VL cases management during subsequent phases of the national program when VL burden is low and hospitalization of VL patients for 3-5-days is now feasible.

## Introduction

Visceral leishmaniasis (VL) or kala-azar has been a public health problem in Bangladesh over the centuries [1]. About 150,000 people have suffered from VL since 1994 in Bangaldesh [2]. Victims of VL are the poorest of the poor living in the rural areas of the country. The epidemiology of VL in Bangladesh, India, and Nepal is similar. In these three countries, the protozoan parasite *Leishmania donovani* (LD) is the only causative agent of VL, humans are the only reservoir, and the female *Phlebotomas argentipes* sandfly is the only vector. In 2005, these three countries signed a memorandum of understanding to eliminate VL as a public health problem by 2015, targeting to keep VL incidence less than 1 per 10 000 people at *upazila* (sub-district), block and district levels in Bangladesh, India and Nepal respectively [3]. However, the target was not achieved in 2015. Current WHO Road Map aims to eliminate Neglected Tropical Diseases including VL by 2020 [4]. Bangladesh and Nepal achieved the target in 2016 and 2013 respectively.

VL peaks periodically in the Indian subcontinent; this is known as the natural trend of VL [5]. It is believed that during inter-epidemic periods, cases of post-kala-azar dermal leishmaniasis (PKDL) fuel the transmission of LD in endemic communities. PKDL is a sequel of infection by LD parasite; it develops mostly among patients who have been treated for VL [6–7]. PKDL also may develop among individuals with asymptomatic infection with the parasite [7]. Hypopigmented macular, papular, and nodular or combined skin lesion are the key clinical features of PKDL [6]. Skin lesions usually do not itch and have intact skin sensitivity. Patients with PKDL are clinically healthy and usually do not seek medical care unless they are stigmatized by their lesions. Patients with PKDL harbor the parasite in their skin for years and are infective to sandflies [8–9]. In this way, they continue transmission of LD in the community and threaten VL control in long run. Failure to control PKDL, therefore, is a substantial challenge to the success of the South-East Asia Region Kala-azar Elimination Programme (KAEP) [10].

Visceral leishmaniasis relapse (VLR) is defined as the reappearance of VL after complete cure at 6 months after treatment. VLR patients have higher concentration of the parasite in their peripheral blood compared to that of new VL patients, are infective to sandflies, sustain transmission of the infection, and threaten VL control [9, 11]. Therefore, VLR also present challenges for controlling VL in the long run. To date, there are no preventive strategies against PKDL and VLR development.

Sodium stibogluconate (SSG) for 6 months or miltefosine (MF) for 84 days are current treatment options for PKDL [10]. Both drugs are toxic, and serious adverse events associated with them are common [6, 12]. Further, efficacy with either treatment does not exceed 90% and relapse after treatment with MF also has been reported [10]. Therefore, a strategy to prevent PKDL is urgently needed. Incomplete treatment for VL has been shown as a risk factor for PKDL in Nepal [13]. A study in India found that VL patients living in areas with high arsenic exposure are more prone to develop PKDL [14]. Immune gene polymorphism has been associated with the development of PKDL in Sudan [15]. An association between SSG treatment for VL and PKDL development has been speculated [16]. However, subsequent reports showed that PKDL also developed after treatment of VL with MF, paromomycin intramuscular injection (PMIM), and liposomal amphotericin B [AmBisome (AMB)] [17–19]. To date, there has been no study of the relationship between treatment for VL and development of PKDL and VLR.

SSG was the only drug for treatment of VL for more than 70 years. Later, MF, PMIM, and AMB either in monotherapy or in combination of any two drugs were introduced for treatment of VL [20–23]. In this study, we aim to investigate the relationship of PKDL and VLR development to VL treatment among cured VL patients treated with different treatment regimens.

## Methods

### Study site, design, population, enrolment, and follow-up of study subjects

The study site was Mymensingh, the most VL-endemic district in Bangladesh. This was a cohort study which included cured VL patients treated with different treatment regimens for new VL (please see below). Duration of the study was October, 2014 to December, 2018. Study participants were enrolled after a study initiation meeting, investigator training, and training of field staff in diagnosis of suspected PKDL and VLR. Using clinical trial logs and hospital records (only for MF cohort), study participants were actively searched and identified at their current places of residence. They were invited to participate in the study to complete four years of follow-up, as well as provide information regarding VLR and PKDL (if any) since treatment for VL. Trained field research assistants followed up study subjects through home visits every 3 months until completion of the 4-year follow-up period. The study physician referred suspected PKDL/VLR cases to the study clinic, Surya Kanta Kala-azar Research Centre, for medical examination, confirmation, and management.

### Study cohort types and sample size

#### SSG cohort

200 new VL patients were treated in the trial “A Pilot Study for Capacity Building for a Multi-centre, Randomized Trial for Treatment of Kala-Azar in Bangladesh,” which was conducted from July 2007 to January 2009 in the Community Based Medical College Hospital, Mymensingh [NCT01240473]. Patients received SSG at a dose of 20 mg/kilogram body weight/day as a single intramuscular injection for 28 days. SSG cohort included 168 cured patients from this trial. This was a completely retrospective cohort; more than 4 years had passed since all participants in this cohort were treated for VL.

#### MF cohort

The National Kala-azar Elimination Programme of Bangladesh (NKEP) used oral MF (Impavido) monotherapy for treatment of new VL from 2009 to mid 2013. MF cohort included 150 cured VL patients who had been diagnosed and treated with MF monotherapy, following the NKEP case management guideline, in the Muktagacha Upazila Health Complex in Mymensingh from April 2010 to July 2013 [24]. Adult patients and children aged 12 years and weighing ≥25 kg received 50 mg MF cap twice/day for 28 days. Children less than 12 years old with body weight <25 kg received oral MF at a dose of 2.5 mg/kilogram/day for 28 days. 60/150 subjects in this cohort had retrospective follow-up; the rest had prospective follow-up.

#### PMIM cohort

The trial titled “Effectiveness study of Paromomycin IM Injection (PMIM) for the treatment of visceral leishmaniasis (VL) in Bangladesh” was a phase IIIb, open-label, multicenter, single-arm trial where 120 new VL patients were treated with PMIM at a dose of 11 mg/kilogram/day for 21 days (NCT01328457) from February 2011 to June 2012 [23]. PMIM cohort had 105 subjects.

#### SDAMB cohort

300 VL patients received treatment with liposomal amphotericin B [AmBisome (AMB)] under an open-label, single-arm trial of a feasibility study (CTRN12612000367842) in the Muktagacha Upazila Health Complex from February 2012 to February 2013 [20]. AMB was applied in a single intravenous infusion at a dose of 10 mg/kilogram body weight for at least 3 hours. SDAMB cohort had 126 subjects.

MDAMB and combination (AMB+PMIM, PMIM+MF, and AMB+MF) cohorts: The study “Safety and efficacy of short course combination regimens with AmBisome, miltefosine and paromomycin for the treatment of visceral leishmaniasis (VL) in Bangladesh” was conducted in the upazila health complexes of Mymensingh from August 2010 to April 2014 (NCT01122771). The study used and compared four treatment regimens for VL [22]. The treatment regimens (and number of patients) were:

- AMB at a dose of 5 mg/kilogram/day on days 1, 3, and 5 (N = 159). For the present study, we assigned 126 cured patients from this regimen to the MDAMB cohort.
- Combination of single intravenous infusion of AMB at a dose of 5mg/kilogram followed by PMIM at 11 mg/kilogram body weight/day (AMB+PMIM) for 10 days (N = 159). AMB+PMIM cohort had 126 cured patients for the current study.
- Combination of single intravenous infusion of AMB at a dose of 5 mg/kilogram on day 1 followed by oral MF (AMB+MF) at above mentioned dosage/day for 7 days (N = 142). We enrolled 126 cured patients from this treatment regimen in the AMB+MF cohort of the present study.
- Combination of PMIM for 10 days and oral MF (PMIM+MF) for 7 days in above mentioned doses (N = 142). We enrolled 126 cured patients from this treatment in the PMIM+MF cohort of the present study.

### Case definitions

A suspected PKDL patient was a person with history of kala-azar and skin lesions; a probable PKDL case was a suspected PKDL case with positive rK39 test; and a confirmed PKDL case was a probable PKDL case with LD parasite documented either by slit skin examination, culture, or polymerase chain reaction. Probable and confirmed PKDL cases were eligible for treatment following the national guideline for kala-azar [24].

A suspected case of VLR was a case of cured VL with fever for more than 2 weeks and splenomegaly. A confirmed case of VLR was a suspected case of VLR with LD bodies in spleen aspirates documented by microscopy/LD DNA in spleen aspirates or peripheral blood buffy coat.

### Statistical analysis

A standard data management plan has been developed by the experts at Clinical data management centre, Christian Medical College (CMC), Vellore, India. Oracle Clinical (OC) version 4.6.6 was used to design web-based dual data entry system following the annotated Case Report Form (CRF) of this study. External monitors checked and approved all CRFs before data entry. Data management team at CMC further scrutinized dual entered data and generated data clarification forms which were clarified by the data management and field team in icddr,b. Following data quality control checks, the data management team at CMC provided a final clean data set for analysis. We used Chi-square test for comparing proportions of various socio-demographic variables of the study participant between different cohorts. Comparison of mean/median between cohorts was done by parametric or non-parametric test where applicable. We calculated both the incidence proportion and rate (100-person-4years) of PKDL and VLR. Finally we performed a Cox-proportional hazard model using SSG cohort with least incidence of PKDL and VLR as reference to investigate the independent risk factor(s) for development of PKDL and VLR. Data were analysis using STATA 13.

### Ethics statement

The icddr,b Ethical Review Committee, Western Institutional Review Board (WIRB), and PATH Research Ethics Committee approved the study. Informed voluntary written consent from adults and assent from children between 11 and 17 years old were obtained for their participation. In cases of study participants who were less than 11 years old, consent from a parent/legal guardian was also obtained.

## Results

### Study population characteristics

974/984 enrolled subjects completed the study, indicating 1% lost to follow-up (Table 1).

**Table 1:** Study profile.

Treatment regimen	Target enrollment	Total enrolled	Not enrolled, % (n)	Reasons for those not enrolled	Follow-up completed	Dropout, % (n)	Reasons for dropout
SSG	200	168	16.0 (32)	Migrated = 16, Not interested = 2, Treatment incomplete = 5, Death = 9,	168	0.0 (0)	
MF	150	150	0.0 (0)		150	0.0 (0)	
PMIM	120	105	12.5 (15)	Migrated = 7, Not interested = 2, Treatment incomplete = 2, Death = 4	105	0.0 (0)	
MDAMB	126	113	10.0 (13)	Migrated = 9, Not interested = 1, Mixed treatment = 1, Death = 1, Pregnant = 1	113	0.0 (0)	
SDAMB	126	126	0.0 (0)		125	0.8 (1)	Migrated
PMIM+MF	126	105	17.0 (21)	Migrated = 13, Not interested = 4, Mixed treatment = 4	101	4.0 (4)	Migrated = 1,Death = 1, Lost to follow-up = 2
AMB+PMIM	126	112	11.0 (14)	Migrated = 13, Not interested = 1	109	3.0 (3)	Migrated = 2, Death = 1
AMB+MF	126	105	17.0 (21)	Migrated = 14, Not interested = 1, Mixed treatment = 2, Death = 3, Pregnant = 1	103	2.0 (2)	Death = 2
*Total*	1100	984	10.5 (116)		974	1.0 (10)	

Study cohorts differed significantly in terms of age groups, sex, education level, monthly expenditure, family size, house type, ownership of cattle and bed-nets, and bed-net use (Table 2).

**Table 2:** Demographic comparison among different treatment regimens.

Indicator	SSG N = 168	MF N = 150	PMIM N = 105	MDAMB N = 113	SDAMB N = 126	PMIM+MF N = 105	AMB+PMIM N = 112	AMB+MF N = 105	Total N = 984
**Age in years, Mean (95% CI)**	28.3 (26.2, 30.3)^**a**^	25.9 (23.1, 28.6)^**b**^	22.5 (20.3, 24.8)^**a**^	24.7 (21.9, 27.5)	19.4 (17.2, 21.6)^**a**^^,^^**b**^^,^^**e**^	21.7 (19.0, 24.3)^**a**^	23.4 (20.7, 26.1)	26.4 (23.5, 29.4)^**e**^	24.3 (23.4, 25.2)
**Age group, % (95% CI) (n)**									
<18 years	28.6 (22.2, 35.9) (48)^**a**^	46.7 (38.8, 54.8) (70)^**a**^^,^^**b**^	45.7 (36.3, 55.4) (48)^**a**^^,^^**b**^^,^^**c**^	45.1 (36.1, 54.5) (51)^**a**^	57.9 (49.0, 66.4) (73)^**a**^^,^^**b**^^,^^**e**^	52.4 (42.7, 61.9) (55)^**a**^	45.5 (36.4, 55.0) (51)^**a**^	41.9 (32.7, 51.7) (44)^**c**^^,^^**e**^	44.7 (41.6, 47.8) (440)
(18–45) years	54.8 (47.1, 62.2) (92)	39.3 (31.8, 47.5) (59)	50.5 (40.9, 60.1) (53)	43.4 (34.4, 52.8) (49)	36.5 (28.5, 45.4) (46)	39.1 (30.1, 48.8) (41)	43.8 (34.7, 53.2) (49)	43.8 (34.5, 53.6) (46)	44.2 (41.1, 47.3) (435)
>45 years	16.7 (11.7, 23.2) (28)	14.0 (9.3, 20.6) (21)	3.8 (1.4, 9.9) (4)	11.5 (6.7, 19.0) (13)	5.6 (2.6, 11.3) (7)	8.6 (4.5, 15.8) (9)	10.7 (6.1, 18.1) (12)	14.3 (8.7, 22.5) (15)	11.1 (9.3, 13.2) (109)
**Sex, % (95% CI) (n)**									
Male	54.8 (47.1, 62.2) (92)^**a**^	57.3 (49.2, 65.1) (86)	61.0 (51.2, 69.9) (64)	58.4 (49.0, 67.3) (66)	61.9 (53.0, 70.1) (78)	65.7 (56.0, 74.3) (69)	56.3 (46.8, 65.3) (63)	68.6 (58.9, 76.8) (72)^**a**^	60.0 (56.9, 63.0) (590)
Female	45.2 (37.8, 52.9) (76)	42.7 (34.9, 50.8) (64)	39.1 (30.1, 48.8) (41)	42.0 (32.8, 51.0) (47)	38.1 (29.9, 47.0) (48)	34.3 (25.7, 44.0) (36)	43.8 (34.7, 53.2) (49)	31.4 (23.2, 41.1) (33)	40.0 (37.0, 43.1) (394)
**Education, % (95% CI) (n)**									
Illiterate	47.0 (39.5, 54.7) (79)	50.7 (42.6, 58.7) (76)^**b**^	39.1 (30.1, 48.8) (41)	38.9 (30.3, 48.4) (44)^**b**^	39.7 (31.4, 48.6) (50)^**b**^	34.3 (25.7, 44.0) (36)^**b**^^,^^**f**^	39.3 (30.6, 48.8) (44)^**b**^^,^^**f**^	44.8 (35.4, 54.5) (47)	42.4 (39.3, 45.5) (417)
Primary level	35.7 (28.8, 43.3) (60)	36.7 (29.3, 44.8) (55)	38.1 (29.2, 47.9) (40)	35.4 (27.0, 44.8) (40)	37.3 (29.2, 46.2) (47)	47.6 (38.1, 57.3) (50)	32.1 (24.1, 41.5) (36)	36.2 (27.5, 46.0) (38)	37.2 (34.2, 40.3) (366)
High school and above	17.3 (12.2, 23.8) (29)	12.7 (8.2, 19.1) (19)	22.9 (15.7, 32.0) (24)	25.7 (18.4, 34.6) (29)	23.0 (16.4, 31.3) (29)	18.1 (11.8, 26.8) (19)	28.6 (20.9, 37.8) (32)	19.1 (12.5, 27.9) (20)	20.4 (18.0, 23.1) (201)
**Monthly expenditure** **(BDT), Mean (95% CI)**	7934.5 (7309.4, 8559.6)^**a**^	7850.0 (7220.1, 8479.9)^**b**^	8742.9 (7981.3, 9504.4)^**c**^	8199.1 (7647.3, 8751.0)	7627.0 (7009.4, 8244.5)^**c**^^,^^**e**^	9071.4 (8279.2, 9863.6)^**a**^^,^^**b**^^,^^**e**^	8732.1 (7871.7, 9592.6)^**e**^	8295.2 (7692.8, 8897.7)	8249.5 (8009.1, 8489.9)
**Family members in the** **HH, Mean (95% CI)**	5.2 (5.0, 5.5)	5.6 (5.3, 5.9)	5.5 (5.1, 5.8)	5.3 (4.9, 5.6)	5.3 (5.0, 5.7)	5.5 (5.1, 5.9)	5.6 (5.2, 6.0)	5.4 (5.0, 5.8)	5.4 (5.3, 5.5)
**Family member affected by** **VL in the past, % (95% CI) (n)**	37.5 (30.4, 45.1) (63)	28.0 (21.3, 35.8) (42)^**b**^	26.7 (19.0, 36.1) (28)^**c**^	38.1 (29.5, 47.5) (43)	34.9 (27.0, 43.8) (44)	41.0 (31.8, 50.7) (43)^**b**^^,^^**c**^	31.3 (23.3, 40.6) (35)	36.2 (27.5, 46.0) (38)	34.2 (31.2, 37.2) (336)
**House type, % (95% CI) (n)**									
*Kuccha mud / thatched)*	27.4 (21.1, 34.7) (46)^**a**^	17.3 (12.0, 24.3) (26)^**a**^^,^^**b**^	41.9 (32.7, 51.7) (44)^**a**^^,^^**b**^	34.5 (26.2, 43.9) (39)^**b**^	30.2 (22.7, 38.8) (38)^**b**^^,^^**e**^	39.1 (30.1, 48.8) (41)^**a**^^,^^**b**^^.^^**e**^	34.8 (26.5, 44.2) (39)^**a**^^,^^**b**^^,^^**e**^^**,g**^	34.3 (25.7, 44.0) (36)^**b**^^,^^**g**^	31.4 (28.6, 34.4) (309)
*Pucca (cemented)*	10.1 (6.4, 15.7) (17)	5.3 (2.7, 10.4) (8)	11.4 (6.5, 19.2) (12)	15.9 (10.2, 24.0) (18)	8.7 (4.9, 15.2) (11)	17.1 (11.0, 25.7) (18)	21.4 (14.7, 30.1) (24)	8.6 (4.5, 15.8) (9)	11.89 (10.0, 14.1) (117)
Tin house	62.5 (54.9, 69.6) (105)	77.3 (69.9, 83.4) (116)	46.7 (37.2, 56.4) (49)	49.6 (40.3, 58.8) (56)	61.1 (52.2, 69.3) (77)	43.8 (34.5, 53.6) (46)	43.8 (34.7, 53.2) (49)	57.1 (47.4, 66.4) (60)	56.7 (53.6, 59.8) (558)
**Having cattle shed,** **% (95% CI) (n)**	54.2 (46.5, 61.6) (91)^**a**^	49.3 (41.3, 57.4) (74)^**b**^	67.6 (58.0, 76.0) (71)^**a**^^,^^**b**^	58.4 (49.0, 67.3) (66)	56.4 (47.5, 64.9) (71)	59.1 (49.3, 68.2) (62)	58.9 (49.5, 67.8) (66)	65.7 (56.0, 74.3) (69)^**b**^	57.9 (54.8, 61.0) (570)
**No. of bednets in the HH,** **Mean (95% CI)**	2.1 (1.9, 2.2)^**a**^	2.5 (2.3, 2.7)^**a**^^,^^**b**^	2.2 (2.0, 2.4)	2.2 (2.0, 2.4)	2.4 (2.2, 2.5)^**a**^	2.1 (1.9, 2.4)^**b**^	2.1 (1.9, 2.4)^**b**^	2.3 (2.1, 2.5)	2.2 (2.2, 2.3)
**Use of bednet,** **% (95%CI) (n)**									
Frequently	63.1 (55.5, 70.1) (106)^**a**^	86.7 (80.2, 91.3) (130)^**a**^^,^^**b**^	67.6 (58.0, 76.0) (71)^**b**^^,^^**c**^	73.5 (64.4, 80.9) (83)^**a**^^,^^**b**^^,^^**c**^^,^^**d**^	80.2 (72.2, 86.3) (101)^**a**^^,^^**d**^^,^^**e**^	64.8 (55.0, 73.4) (68)^**b**^^,^ ^**e**^	66.1 (56.7, 74.3) (74)^**b**^^,^ ^**e**^	62.9 (53.1, 71.7) (66)^**a**^^,^^**b**^^,^^**e**^	71.0 (68.1, 73.8) (699)
Sometimes	31.0 (24.4, 38.4) (52)	10.7 (6.6, 16.8) (16)	29.5 (21.5, 39.1) (31)	18.6 (12.4, 27.0) (21)	19.1 (13.0, 27.0) (24)	27.6 (19.8, 37.1) (29)	28.6 (20.9, 37.8) (32)	31.4 (23.2, 41.1) (33)	24.2 (21.6, 27.0) (238)
Not at all	0.6 (0.1, 4.2) (1)	0.0 (0)	0.0 (0)	4.4 (1.8, 10.3) (5)	0.0 (0)	1.0 (0.1, 6.7) (1)	1.8 (0.4, 7.0) (2)	4.8 (2.0, 11.1) (5)	1.4 (0.8, 2.4) (14)
N/A	5.4 (2.8, 10.0) (9)	2.7 (1.0, 7.0) (4)	2.9 (0.9, 8.6) (3)	3.5 (1.3, 9.2) (4)	0.8 (0.1, 5.6) (1)	6.7 (3.2, 13.5) (7)	3.6 (1.3, 9.3) (4)	1.0 (0.1, 6.7) (1)	3.4 (2.4, 4.7) (33)

AMB = AmBisome. BDT = Bangladeshi Taka. HH = household. MDAMB = multidose liposomal amphotericin B (AmBisome). MF = miltefosine. N/A = not applicable. No. = number. PMIM = paromomycin intramascular injection. SDAMB = single-dose liposomal amphotericin B (AmBisome). SSG = sodium stibogluconate. VL = visceral leishmaniasis.

^a^ indicates SSG versus others with p<0.05.

^b^ indicates MF versus others with p<0.05.

^c^ indicates PMIM versus others with p<0.05.

^d^ indicates MDAMB versus others with p<0.05.

^e^ indicates SDAMB versus others with p<0.05.

^f^ indicates PMIM+MF versus others with p<0.05.

^g^ indicates AMB+PMIM versus others with p<0.05.

These variables were taken as covariates for multivariate analysis later.

### Burden, risk factor, and trend for PKDL

121/984 developed PKDL (mean, 95% CI, 12.3%, 10.4%–14.5%) with a median time 2.6 years (IQR, 1.84–3.12). The incidence proportion (mean, 95% CI) with PKDL was lowest in the SSG (3.0%, 1.2%–7.0%), followed by the MDAMB (8.0%, 4.2%–14.7%), MF (9.3%, 5.6%–15.2%), AMB+PMIM (10.7%, 6.1%–18.1%), SDAMB (15.9%, 10.4%–23.5%), AMB+MF (16.2%, 10.2%–24.7%), PMIM (19.1%, 12.5%–27.9%), and PMIM+MF (22.9%, 15.7%–32.0%) (Table 3). The SSG differed significantly from all other cohorts, except the MDAMB cohort.

**Table 3:** Incidence of PKDL by treatment regimens.

Treatment regimen	No. of participants	PKDL cases	Total survival time (in days)	Mean (95%CI) survival time (in days)	Incidence rate in 100-person-years for 4 years (95% CI)	Incidence proportion for 4 years % (95% CI)
SSG	168	5	242 072	1440.9 (1423.0, 1458.8)	3.0 (1.3, 7.3)^a^	3.0 (1.2, 7.0)^a^
MDAMB	113	9	160 709	1422.2 (1394.9, 1449.5)	8.2 (4.3, 15.7)^b^	8.0 (4.2, 14.7)^b^
MF	150	14	210 810	1405.4 (1374.4, 1436.4)	9.7 (5.7, 16.4)^a^^,^^c^	9.3 (5.6, 15.2)^a^^,^^c^
AMB+PMIM	112	12	155 391	1387.4 (1348.3, 1426.6)	11.3 (6.4, 19.9)^a^^,^^d^	10.7 (6.1, 18.1)^a^^,^^d^
SDAMB	126	20	172 688	1370.5 (1328.7, 1412.4)	16.9 (10.9, 26.2)^a^	15.9 (10.4, 23.5)^a^
AMB+MF	105	17	145 321	1384.0 (1345.6, 1422.5)	17.1 (10.6, 27.5)^a^	16.2 (10.2, 24.7)^a^
PMIM	105	20	144 984	1380.8 (1338.1, 1423.5)	20.1 (13.0, 31.2)^a^^,^^b^^,^^c^	19.1 (12.5, 27.9)^a^^,^^b^^,^^c^
PMIM+MF	105	24	138 653	1320.5 (1264.6, 1376.5)	25.3 (16.9, 37.7)^a^^,^^b^^,^^c^^,^ ^d^	22.9 (15.7, 32.0)^a^^,^^b^^,^^c^^,^ ^d^
Total	984	121	1 370 628	1392.9 (1380.1, 1405.7)	14.0 (8.6, 22.7)	12.3 (10.4, 14.5)

AMB = AmBisome. MDAMB = multidose liposomal amphotericin B (AmBisome). MF = miltefosine. No. = number. PKDL = post-kala-azar dermal leishmaniasis. PMIM = paromomycin intramascular injection. SDAMB = single-dose liposomal amphotericin B (AmBisome). SSG = sodium stibogluconate.

^a^ indicates SSG versus others with p<0.05.

^b^ indicates MDAMB versus others with p<0.05.

^c^ indicates MF versus others with p<0.05.

^d^ indicates AMB+PMIM versus others with p<0.05.

All 984 participants contributed 1 370 628 days of observation, with a mean of 1393 days (95% CI, 13 80–1406) when cohorts were looked for PKDL development. The average incidence rate (100-person-4years) of PKDL was 14.0 (8.6–22.7). SSG cohort had lowest incidence rate (3.0, 1.3–7.3), followed by the MDAMB (8.2, 4.3–15.7), MF (9.7, 5.7–16.4), AMB+PMIM (11.3, 6.4–19.9), SDAMB (16.9, 10.9–26.2), AMB+MF (17.1, 10.6–27.5), PMIM (20.1, 13.0–31.2), and PMIM+MF (25.3, 16.9–37.7) (Table 3). Incidence rate of PKDL of SSG and MDAMB cohorts did not differ significantly.

Assuming SSG cohort as a reference we analyzed Cox Proportional Hazard Ratio for other cohorts for development of PKDL (Table 4). The average (95% CI) hazard ratio adjusted for confounders for development of PKDL was 2.7 (0.9–8.1), 3.4 (1.2–9.5), 3.5 (1.2–10.0), 5.8 (2.1–15.8), 6.0 (2.2–16.4), 6.2 (2.3–16.7), and 8.0 (3.0–21.1) for MDAMB, MF, AMB+PMIM, SDAMB, AMB+MF, PMIM, and PMIM+MF, respectively (Table 4). The MDAMB’s hazard ratio did not differ statistically significantly with the reference cohort. However, hazard ratio of all other cohorts was statistically significant (Table 4). We did not find any covariates as a statistically significant factor for PKDL development (Table 4). Further, when compared existing treatment regimens excluding SSG and using MDAMB as a reference, all treatment regimens had higher hazard ratio for PKDL which was statistically significant for AMB+MF, PMIM and PMIM+MF (Table 5), borderline significant for SDAMB and statistically insignificant MF and AMB+PMIM (Table 5).

**Table 4:** Cox proportional hazard regression by treatment regimen for PKDL.

Treatment regimen	Unadjusted hazard ratio (95% CI)	P-value	Adjusted hazard ratio (95% CI)^a^	P-value
SSG	1	—	1	—
MDAMB	2.7 (0.9, 8.1)	0.07	2.7 (0.9, 8.1)	0.09
MF	3.3 (1.2, 9.0)	0.02	3.4 (1.2, 9.5)	0.02
AMB+PMIM	3.8 (1.3, 10.8)	0.01	3.5 (1.2, 10.0)	0.02
SDAMB	5.7 (2.2, 15.3)	<0.0001	5.8 (2.1, 15.8)	0.001
AMB+MF	5.8 (2.1, 15.6)	0.001	6.0 (2.2, 16.4)	<0.0001
PMIM	6.8 (2.6, 18.1)	<0.0001	6.2 (2.3, 16.7)	<0.0001
PMIM+MF	8.7 (3.3, 22.9)	<0.0001	8.0 (3.0, 21.1)	<0.0001

AMB = AmBisome. MDAMB = multidose liposomal amphotericin B (AmBisome). MF = miltefosine. No. = number. PKDL = post-kala-azar dermal leishmaniasis. PMIM = paromomycin intramascular injection. SDAMB = single-dose liposomal amphotericin B (AmBisome). SSG = sodium stibogluconate. VL = visceral leishmaniasis.

^a^ Adjusted covariates: Age, Gender, Education, Monthly household expenditure, Number of family members in the household, Family member affected by VL in the past, Type of house, Presence of cattle shed, Number of bednets in the household, How often is the bednet used.

**Table 5:** Cox proportional hazard regression by treatment regimen for PKDL; MDAMB as a reference group.

Treatment regimen	Unadjusted hazard ratio (95% CI)	P-value	Adjusted hazard ratio (95% CI)^a^	P-value
MDAMB	1	—	1	—
MF	1.2 (0.5, 2.8)	0.67	1.2 (0.5, 2.9)	0.62
AMB+PMIM	1.4 (0.6, 3.3)	0.45	1.2 (0.5, 2.9)	0.63
SDAMB	2.1 (0.9, 4.6)	0.06	2.0 (0.9, 4.4)	0.08
AMB+MF	2.1 (0.9, 4.7)	0.06	2.3 (1.0, 5.2)	0.04
PMIM	2.5 (1.1, 5.5)	0.02	2.3 (1.0, 5.0)	0.04
PMIM+MF	3.2 (1.5, 6.9)	0.003	3.0 (1.4, 6.5)	0.005

AMB = AmBisome. MDAMB = multidose liposomal amphotericin B (AmBisome). MF = miltefosine. No. = number. PKDL = post-kala-azar dermal leishmaniasis. PMIM = paromomycin intramascular injection. SDAMB = single-dose liposomal amphotericin B (AmBisome). VL = visceral leishmaniasis.

^a^ Adjusted covariates: Age, Gender, Education, Monthly household expenditure, Number of family members in the household, Family member affected by VL in the past, Type of house, Presence of cattle shed, Number of bednets in the household, How often is the bednet used.

**Fig 1:**
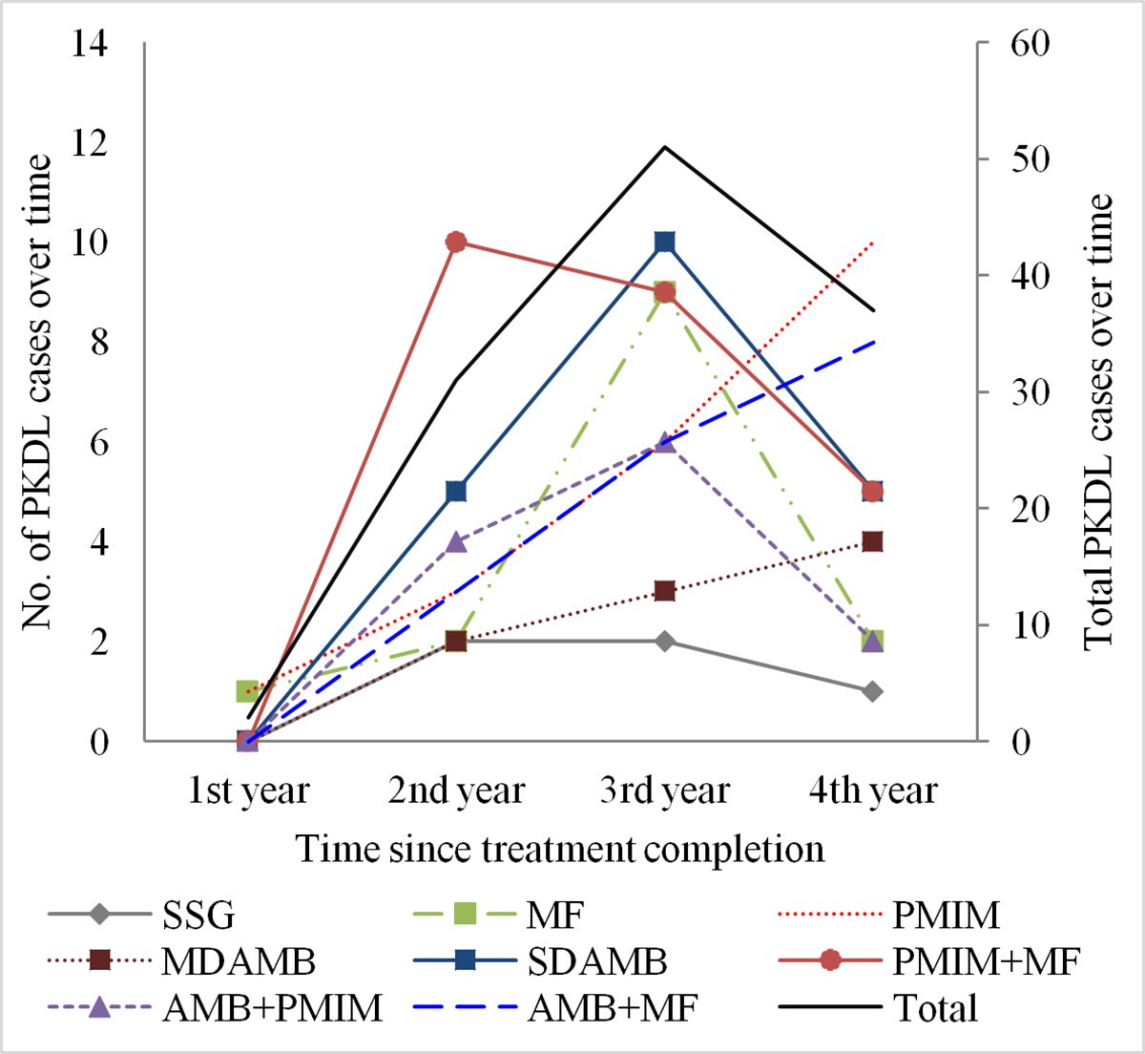
Trend of PKDL case development by treatment regimen.

The average trend for development of PKDL peaked at year 3 since treatment for VL (Fig 1). Interestingly, when stratified by cohorts, the PMIM, AMB+MF, and MDAMB cohorts showed upward trends for PKDL development (Fig 1).

### Burden, risk factor, and trend for VLR

Of the 984 participants, 69 had VLR with a median time 1.05 years (IQR, 0.77–1.53) for 1 364 974 person-days of observation (mean 95% CI, 1387, 1371–1404) (Table 6).

**Table 6:** Incidence of VL relapse by treatment regimens.

Treatment regimen	No. of participants	VLR cases	Total survival time (in days)	Mean (95%CI) survival time (in days)	Incidence rate in 100-person-years for 4 years (95% CI)	Incidence proportion for 4 years % (95% CI)
SSG	168	3	244 162	1453.4 (1444.4, 1462.3)	1.8 (0.6, 5.6)^a^	1.8 (0.6, 5.5)^a^
MDAMB	113	3	161 885	1432.6 (1401.4, 1463.8)	2.7 (0.9, 8.4)^b^	2.7 (0.8, 8.0)^**b**^
AMB+PMIM	112	4	158 929	1419.0 (1381.2, 1456.8)	3.7 (1.4, 9.8)^c^	3.6 (1.3, 9.3)^**c**^
PMIM+MF	105	5	146 218	1392.6 (1347.2, 1437.9)	5.0 (2.1, 12.0)^d^	4.8 (2.0, 11.1)^**d**^
AMB+MF	105	7	145 819	1388.8 (1338.2, 1439.4)	7.0 (3.3, 14.7)^a^^,^^e^	6.7 (3.2, 13.5)^a^^,^^**e**^
SDAMB	126	10	173 535	1377.3 (1326.5, 1428.1)	8.4 (4.5, 15.6)^a^	7.9 (4.3, 14.2)^a^
PMIM	105	15	137 225	1306.9 (1232.6, 1381.2)	16.0 (9.6, 26.5)^a^^,^^b^^,^^c^^,^^d^	14.3 (8.7, 22.5)^a^^,^^b^^,^^c^^,^^d^
MF	150	22	197 201	1314.7 (1255.2, 1374.1)	16.3 (10.7, 24.7)^a^^,^^b^^,^^c^^,^^d^^,^^e^	14.7 (9.8, 21.4)^a^^,^^b^^,^^c^^,^^d^^,^^**e**^
Total	984	69	1 364 974	1387.2 (1370.5, 1403.8)	7.6 (4.1, 14.7)	7.0 (5.6, 8.8)

AMB = AmBisome. MDAMB = multidose liposomal amphotericin B (AmBisome). MF = miltefosine. No. = number. PKDL = post-kala-azar dermal leishmaniasis. PMIM = paromomycin intramascular injection. SDAMB = single-dose liposomal amphotericin B (AmBisome). SSG = sodium stibogluconate. VL = visceral leishmaniasis. VLR = visceral leishmaniasis relapse.

^a^ indicates SSG versus others with p<0.05.

^b^ indicates MDAMB versus others with p<0.05.

^c^ indicates AMB+PMIM versus others with p<0.05.

^d^ indicates PMIM+MF versus others with p<0.05.

^e^ indicates AMB+MF versus others with p<0.05.

Overall incidence proportion of VLR was 7% (95% CI, 7.0%, 5.6%–8.8%). The SSG cohort had lowest incidence proportion (mean, 95% CI) for VLR (1.8%, 0.6%–5.5%), followed by the MDAMB (2.7%, 0.8%–8.0%), AMB+PMIM (3.6%, 1.3%–9.3%), PMIM+MF (4.8%, 2.0%–11.1%), AMB+MF (6.7%, 3.2%–13.5%), SDAMB (7.9%, 4.3%–14.2%), PMIM (14.3%, 8.7%–22.5%), and MF arms (14.7%, 9.8%–21.4%).

**Fig 2:**
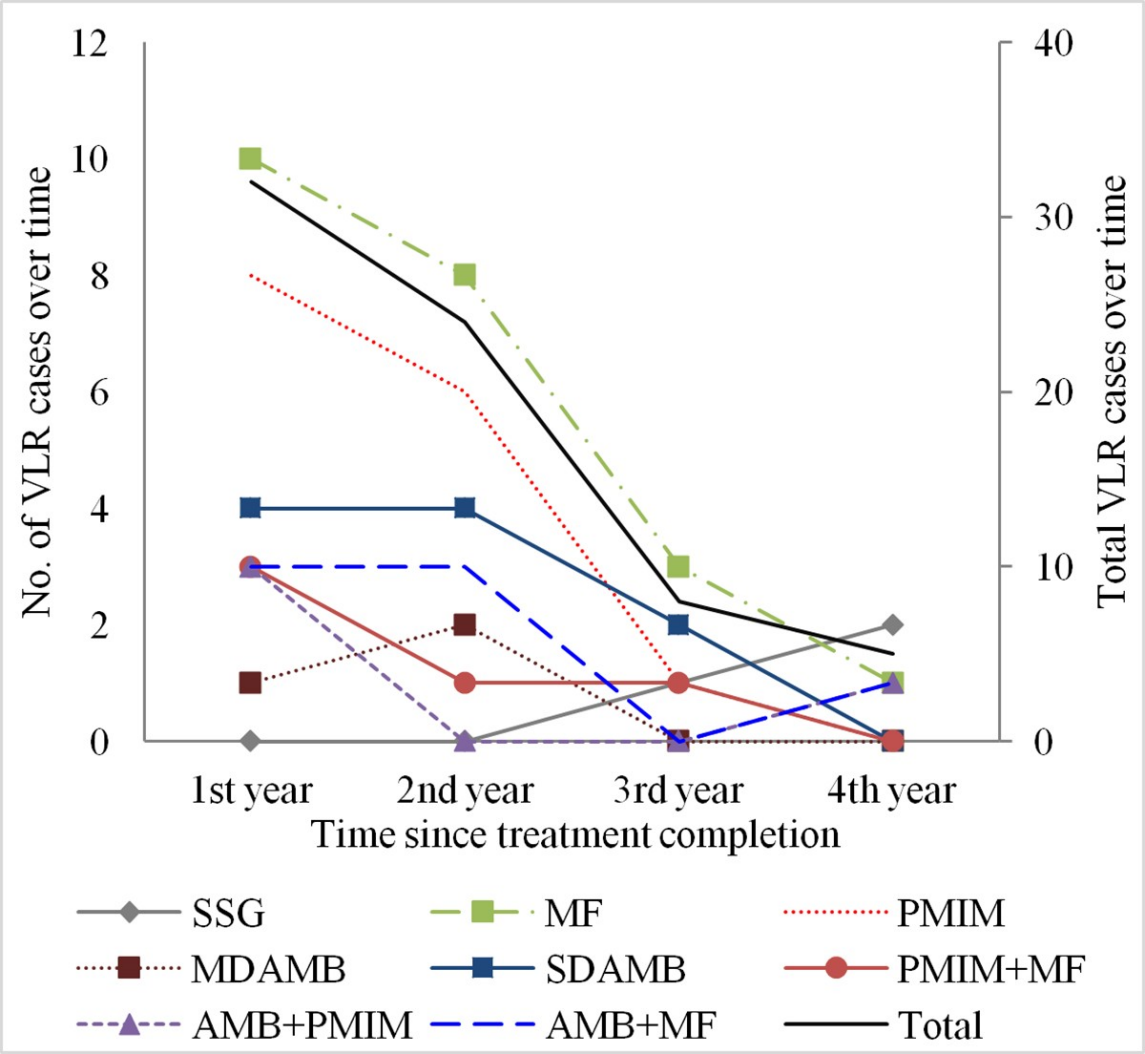
Trend of VLR case development by treatment regimen.

The overall incidence rate (100-person-4years) of VLR was 7.6 (95% CI, 4.1–14.7). SSG cohort had lowest VLR incidence rate (rate, 95%CI) (1.8, 0.6–5.6), followed by the MDAMB (2.7, 0.9–8.4), AMB+PMIM (3.7, 1.4–9.8), PMIM+MF (5.0, 2.1–12.0) AMB+MF (7.0, 3.3–14.7), SDAMB (8.4, 4.5–15.6), PMIM (16.0, 9.6–26.5), and MF (16.3, 10.7–24.7) cohorts (Table 6). The Cox proportional hazard ratio (mean, 95% CI) for VLR incidence adjusted for covariates considering the SSG arm as reference, was lowest for the MDAMB arm (1.3, 0.3–6.3), followed by the AMB+PMIM (1.9, 0.4–8.7), PMIM+MF (2.2, 0.5–9.2), AMB+MF (3.5, 0.9–13.5), SDAMB (3.5, 1.0–13.0), MF (7.5, 2.2–25.6), and PMIM (7.7, 2.2–27.1) cohorts (Table 7). The hazard ratio of the MDAMB, AMB+PMIM, PMIM+MF, AMB+MF, and SDAMB arms for VLR was higher but statistically insignificant, but the higher hazard ratio of the MF and PMIM arms was statistically significant (Table 7). None of the covariates had any significant association with VLR development. Using MDAMB as a reference we found higher but statistically insignificant hazard ration for VLR of AMB+PMIM, PMIM+MF, AMB+MF and SDAMB whereas hazard ration for VLR of MF and PMIM was six times higher and statistically significant (Table 8). The overall trend of VLR peaked at 1 year and then declined thereafter. The VLR trend had a similar pattern for all study arms (Fig 2).

**Table 7:** Cox proportional hazard regression by treatment regimen for VLR.

Treatment regimen	Unadjusted hazard ratio (95% CI)	P-value	Adjusted hazard ratio (95% CI)^a^	P-value
SSG	1	—	1	—
MDAMB	1.5 (0.3, 7.5)	0.62	1.3 (0.3, 6.3)	0.78
AMB+PMIM	2.0 (0.5, 9.1)	0.35	1.9 (0.4, 8.7)	0.39
PMIM+MF	2.7 (0.7, 11.4)	0.17	2.2 (0.5, 9.2)	0.30
AMB+MF	3.9 (1.0, 14.9)	0.05	3.5 (0.9, 13.5)	0.07
SDAMB	4.6 (1.3, 16.8)	0.02	3.5 (1.0, 13.0)	0.06
MF	8.9 (2.7, 29.6)	<0.0001	7.5 (2.2, 25.6)	0.001
PMIM	8.7 (2.5, 30.0)	0.001	7.7 (2.2, 27.1)	0.001

AMB = AmBisome. MDAMB = multidose liposomal amphotericin B (AmBisome). MF = miltefosine. PMIM = paromomycin intramascular injection. SDAMB = single-dose liposomal amphotericin B (AmBisome). SSG = sodium stibogluconate. VL = visceral leishmaniasis. VLR = visceral leishmaniasis relapse.

^a^ Adjusted covariates: Age, Gender, Education, Monthly household expenditure, Number of family members in the household, Family member affected by VL in the past, Type of house, Presence of cattle shed, Number of bednets in the household, How often is the bednet used.

**Table 8:** Cox proportional hazard regression by treatment regimen for VLR; MDAMB as a reference group.

Treatment regimen	Unadjusted hazard ratio (95% CI)	P-value	Adjusted hazard ratio (95% CI)^a^	P-value
MDAMB	1	—	1	—
AMB+PMIM	1.4 (0.3, 6.1)	0.68	1.5 (0.3, 6.6)	0.61
PMIM+MF	1.8 (0.4, 7.6)	0.41	1.6 (0.4, 6.7)	0.53
AMB+MF	2.6 (0.7, 9.9)	0.17	2.9 (0.7, 11.4)	0.12
SDAMB	3.1 (0.8, 11.2)	0.08	2.7 (0.7, 9.9)	0.13
MF	5.9 (1.8, 19.7)	0.004	6.2 (1.8, 21.0)	0.004
PMIM	5.8 (1.7, 20.0)	0.006	6.1 (1.7, 21.2)	0.005

AMB = AmBisome. MDAMB = multidose liposomal amphotericin B (AmBisome). MF = miltefosine. PMIM = paromomycin intramascular injection. SDAMB = single-dose liposomal amphotericin B (AmBisome). VL = visceral leishmaniasis. VLR = visceral leishmaniasis relapse.

^a^ Adjusted covariates: Age, Gender, Education, Monthly household expenditure, Number of family members in the household, Family member affected by VL in the past, Type of house, Presence of cattle shed, Number of bednets in the household, How often is the bednet used.

## Discussion

Key findings of the current study are: there was a significant relationship between the treatment regimens for VL and the development of PKDL and VLR; and the socio-demographic factors investigated in this study did not have any relationship with PKDL nor with VLR development. The study is unique and included all treatment regimens so far for VL.

SSG was the only treatment for VL for a century. It became less efficacious over time due to parasite resistance to SSG. Further, the World Health Organization (WHO) Expert Committee on the Control of Leishmaniases (WHOECCL) does not recommend SSG for VL due to its long treatment duration and severe toxicity [25]. Though SSG for VL was protective against PKDL and VLR, we do not recommend this treatment for VL due to its severe toxicity. The SSG cohort, however, served as reference—as a cohort with the lowest burden of PKDL and VLR and facilitated comparison of PKDL and VLR by other treatment regimens. In our study, SSG resulted in 3.0% (95% CI, 1.3–7.3) incidence of PKDL. A study in Nepal reported a little higher incidence of PKDL after SSG treatment for VL at 5.4%. It also found incomplete treatment for VL as a risk factor for PKDL.^13^ The difference between the two studies can be explained by the difference in study designs and populations. In our study, all patients had complete treatment with SSG; this was not case in the Nepalese study. A population-based study in Bangladesh found a cumulative incidence of PKDL of 17% for 5 years [7]. The study had 1002 VL patients treated with SSG, MF, and AMB. The study did not stratify PKDL incidence by treatment regimens.

MF monotherapy was introduced in the KAEP after successful completion of phase III and phase IV studies [26–27]. Decreased efficacy of MF; high rate of treatment incompliance; adverse reactions, including renal toxicity and hepatotoxicity; and the availability more safe and effective drug (AMB), led the WHOECCL not to recommend MF monotherapy for treatment of VL.^25^ The highest rate of VLR after MF monotherapy in this study (16.3%), further justify the recommendation of the WHOECCL [25].

PMIM monotherapy was developed by the One World Health for treatment of VL [23]. We observed very high rates of PKDL (20%) and VLR (16%) with this treatment for VL. Our observation supports the WHOECCL, which did not recommend PMIM monotherapy for VL [25].

Availability of AMB, a highly safe and effective drug for treatment of VL, changes the scenario for VL case management. Currently, this is the drug of choice for VL case management in the KAEP. AMB is expensive; the national programs of Bangladesh, India, and Nepal get it as a donation from the developer Gilead Sciences, Inc. through WHO.

The KAEP has three phases: the attack phase, consolidation phase, and maintenance phase [24]. During the attack phase, the VL case burden was 21 times higher than the VL elimination target [28]. Bangladesh and Nepal completed the attack phase and achieved the target. AMB in a single intravenous infusion at a dose of 10 mg/kilogram body weight is the first treatment option for VL in Bangladesh. This treatment regimen was particularly suitable in the attack phase due to its high safety and efficacy for VL, 100% compliance, and 1 to 2 days of patient hospitalization. During the attack phase of the NKEP when VL burden was very high, SDAMB was the most suitable VL treatment option. However, our study highlighted a concern about its continuation during the consolidation and maintenance phases of the program, as it resulted in a very high incidence of PKDL (17%) and VLR (8.4%).

Combination therapy with AMB+MF, AMB+PMIM, and PMIM+MF was introduced for treatment of VL by the Drugs for Neglected Diseases *initiative*. Present study provides important findings for the first time that PKDL and VLR also develop after different combination therapies for VL. The AMB+MF and PMIM+MF combinations resulted in a very high incidence of PKDL, 17% and 25% respectively. The combination of AMB+PMIM gave better results; the rate of PKDL and VLR was 11.3% and 3.7% respectively. Among the combination regimens, AMB+PMIM had the least incidence of PKDL and VLR. It is interesting that combination therapies showed different patterns in terms of PKDL and VLR development.

The MDAMB treatment regimen for VL had the lowest incidence of PKDL and VLR compared to all other treatment regimens except SSG. MDAMB resulted in 8.2% PKDL and 2.7% VLR for 4 years. The less incidence of PKDL by MDAMB compared to that by SDAMB could be explained by the findings from a recent experimental study. The study found that the skin pharmacokinetics of AmBisome was different when AmBisome was given as a single dose and as a multidose for treatment of murine cutaneous leishmaniasis [29]. AmBisome when it was given in a multidose it resulted in a better accumulation of the drug in the skin, more reduction in skin parasite load and skin lesion size [29]. A study in India reported even less incidence of PKDL after treatment of VL with MDAMB at a dose of 20 mg/kilogram body weight. Our MDAMB cohort received 15 mg/kilogram body weight of AMB for treatment of their VL. The study in India had PKDL cases who passively reported to the health facility. Therefore, there may have been under-reporting; this could be another reason for the lower PKDL incidence in that study [30].

All cohorts showed a similar trend for VLR: VLR peaked in the first year after treatment. However, PKDL development peaked in the third year after treatment for VL, but this was not the case when results were stratified by treatment regimens. The PKDL trend continued upward with MDAMB, PMIM, and AMB+MF. This necessitates follow-up of cured VL patients for at least 3 years by the NKEP for early detection of PKDL and VLR cases. This also demands longer observation MDAMB, PMIM, and AMB+MF cohorts to find the moment of a downward trend.

Our study has some limitations. The entire SSG and partial MF arms were retrospective cohorts, whereas all other arms were prospective cohorts. The SSG and MF arms were conducted when the VL burden was comparatively higher in the country. Since PKDL and VLR are consequences of VL, it would bias study results if the study aimed to survey the PKDL/VLR burden in the community. Our study aimed to investigate the PKDL/VLR incidence only in cured VL patients; therefore, results are free from biases related to time. Another limitation of the study is that the mean days of observation differed in the arms due to different sample sizes. Therefore, a cohort with higher mean days of observation should have a higher incidence of PKDL/VLR. Interestingly, the SSG arm had the highest mean days of observation and the least incidence of PKDL and VLR. The treatment regimen therefore dictated the incidence of PKDL and VLR. The study had been carried out using cured VL patients of the clinical trials in Bangladesh. So, external validity of the study is yet to be established and its results may not be generalizable for other countries.

In summary, SSG and MDAMB for VL had least incidences of PKDL and VLR. MDAMB had least hazard ratio for PKDL development compare to other treatment regimens. Since SSG is no more recommended for VL, MDAMB should be the choice for VL in the consolidation and maintenance phases of the NKEP in Bangladesh until better molecules than AMB are found. Therefore, we highly recommend MDAMB for treatment of VL for NKEP in Bangladesh during its consolidation and maintenance phases.

## Supporting information

S1 Checklist STROBE checklist. (DOC)
