# Supplementary material for: Relationship between treatment regimens for visceral leishmaniasis and development of post-kala-azar dermal leishmaniasis and visceral leishmaniasis relapse: A cohort study from Bangladesh
Source: PLoS Negl Trop Dis. 2019 Aug 15;13(8):e0007653. doi: 10.1371/journal.pntd.0007653 (PMC6711542; doi:10.1371/journal.pntd.0007653)
Supplement: S1 Checklist — (DOC) [file pntd.0007653.s001.doc]

S1 Checklist: STROBE checklist

|  | Item No. | Recommendation | Page  No. | Relevant text from manuscript |
| --- | --- | --- | --- | --- |
| **Title and abstract** | 1 | (*a*) Indicate the study’s design with a commonly used term in the title or the abstract | 1 | Please see the title of the manuscript |
|  |  | (*b*) Provide in the abstract an informative and balanced summary of what was done and what was found | 2 | Paragraph 2, 3, 4 and 5 |
| Introduction | | | |  |
| Background/rationale | 2 | Explain the scientific background and rationale for the investigation being reported | 5 & 6 | Paragraph 2, 3, 4 and 5 |
| Objectives | 3 | State specific objectives, including any prespecified hypotheses | 6 | Last paragraph |
| Methods | | | |  |
| Study design | 4 | Present key elements of study design early in the paper | 7 | First paragraph of the method section |
| Setting | 5 | Describe the setting, locations, and relevant dates, including periods of recruitment, exposure, follow-up, and data collection | 7 | Method section: First paragraph |
| Participants | 6 | (*a*) *Cohort study*—Give the eligibility criteria, and the sources and methods of selection of participants. Describe methods of follow-up  *Case-control study*—Give the eligibility criteria, and the sources and methods of case ascertainment and control selection. Give the rationale for the choice of cases and controls  *Cross-sectional study*—Give the eligibility criteria, and the sources and methods of selection of participants | 7, 8, 9 | Section: Study cohort type and sample size. Paragraph 1, 2, 3, 4, 5 and 6 |
|  |  | (*b*) *Cohort study*—For matched studies, give matching criteria and number of exposed and unexposed  *Case-control study*—For matched studies, give matching criteria and the number of controls per case | Not applicable |  |
| Variables | 7 | Clearly define all outcomes, exposures, predictors, potential confounders, and effect modifiers. Give diagnostic criteria, if applicable | 9 & 10 | Statistical consideration: Paragraph 1 |
| Data sources/ measurement | 8* | For each variable of interest, give sources of data and details of methods of assessment (measurement). Describe comparability of assessment methods if there is more than one group | 9 & 10 | Statistical consideration |
| Bias | 9 | Describe any efforts to address potential sources of bias | 9, 10, 12 | Statistical consideration & Paragraph 1 of results section |
| Study size | 10 | Explain how the study size was arrived at | 7, 8 and 9 | Paragraph 1-6 under section cohort type and sample size |

Continued on next page

| Quantitative variables | 11 | Explain how quantitative variables were handled in the analyses. If applicable, describe which groupings were chosen and why | 7, 8, 9 and 10 | Dependent groups were PKDL/VLR and independent groups were different cohorts stratified by their treatment for VL. |
| --- | --- | --- | --- | --- |
| Statistical methods | 12 | (*a*) Describe all statistical methods, including those used to control for confounding | 10 | First paragraph under statistical consideration section |
|  |  | (*b*) Describe any methods used to examine subgroups and interactions | Not applicable |  |
|  |  | (*c*) Explain how missing data were addressed | Not applicable |  |
|  |  | (*d*) *Cohort study*—If applicable, explain how loss to follow-up was addressed  *Case-control study*—If applicable, explain how matching of cases and controls was addressed  *Cross-sectional study*—If applicable, describe analytical methods taking account of sampling strategy | 10 | We calculated both the incidence proportion and rate (100-person-4years) of PKDL and VLR. Please see section statistical consideration |
|  |  | (*e*) Describe any sensitivity analyses | Not applicable |  |
| Results | | | | |
| Participants | 13* | (a) Report numbers of individuals at each stage of study—eg numbers potentially eligible, examined for eligibility, confirmed eligible, included in the study, completing follow-up, and analysed | 10 & 11 | 1^st^ paragraph one under result section |
|  |  | (b) Give reasons for non-participation at each stage | 11 | Table 1 |
|  |  | (c) Consider use of a flow diagram |  | Paragraph one under study population and characteristics. Instead of a flow chart we preferred to present this in a table (Table 1) |
| Descriptive data | 14* | (a) Give characteristics of study participants (eg demographic, clinical, social) and information on exposures and potential confounders | 10 & 11 | Table 2 |
|  |  | (b) Indicate number of participants with missing data for each variable of interest | Not applicable |  |
|  |  | (c) *Cohort study*—Summarise follow-up time (eg, average and total amount) | 14 & 18 | 2^nd^ Paragraph under section “Burden, risk factor, and trend for PKDL” and 1^st^ paragraph under section “Burden, risk factor, and trend for VLR” |
| Outcome data | 15* | *Cohort study*—Report numbers of outcome events or summary measures over time | 14 & 18 | 1^st^ paragraphs under section Burden, risk factor, and trend for PKDL” and “Burden, risk factor, and trend for VLR” |
|  |  | *Case-control study—*Report numbers in each exposure category, or summary measures of exposure |  |  |
|  |  | *Cross-sectional study—*Report numbers of outcome events or summary measures |  |  |
| Main results | 16 | (*a*) Give unadjusted estimates and, if applicable, confounder-adjusted estimates and their precision (eg, 95% confidence interval). Make clear which confounders were adjusted for and why they were included | 14, 17, 18, 21 | 3^rd^ Paragraph under section “Burden, risk factor, and trend for PKDL” and 2^nd^ paragraph under section “Burden, risk factor, and trend for VLR”. Table 4 & Table 6 |
|  |  | (*b*) Report category boundaries when continuous variables were categorized | Not applicable |  |
|  |  | (*c*) If relevant, consider translating estimates of relative risk into absolute risk for a meaningful time period | 17 & 21 | We used Cox proportional Hazard Ratio to address this. Table 4 & Table 6 |

Continued on next page

| Other analyses | 17 | Report other analyses done—eg analyses of subgroups and interactions, and sensitivity analyses | Not applicable |  |
| --- | --- | --- | --- | --- |
| Discussion | | | | |
| Key results | 18 | Summarise key results with reference to study objectives | 22 | 1^st^ paragraph of discussion section |
| Limitations | 19 | Discuss limitations of the study, taking into account sources of potential bias or imprecision. Discuss both direction and magnitude of any potential bias | 25 | 1^st^ Paragraph |
| Interpretation | 20 | Give a cautious overall interpretation of results considering objectives, limitations, multiplicity of analyses, results from similar studies, and other relevant evidence | 25 | Last paragraph |
| Generalisability | 21 | Discuss the generalisability (external validity) of the study results | 25 | 10^th^ Paragraph of discussion |
| Other information | |  | | |
| Funding | 22 | Give the source of funding and the role of the funders for the present study and, if applicable, for the original study on which the present article is based | 2 | Bill and Melinda Gates Foundation |
